# Communications from the Archives of the Society of Physicians Practising in Riga

**Published:** 1841-10

**Authors:** 


### Art. VIII.

*Mittheilungen aus dem Archiv der Gesellschaft practischer Aerzte zu Riga.* Erste Sammlung.—*Leipzig, Riga, und Mitau,* 1839. 8vo, pp. 202.

*Communications from the Archives of the Society of Physicians practising in Riga.* First Collection.—*Leipsig, Riga, and Mitau,* 1839.

This small volume is one of a class which merits especial attention from reviewers, as it is an indication and proof that the members of the profession where it originates are zealous and intelligent beyond the ordinary standard. It is an easy matter for the medical associations of large cities, with a long list of members, and these only the elect of a body of resident practitioners vastly more numerous still, to collect memoirs and publish transactions; but when the same is done by the physicians and surgeons of towns having only a small population, the slightest consideration justifies the inference above stated respecting the character of the resident medical staff. The population of Riga is under 40,000, and it can boast of no important institutions, especially calculated to foster medical science. The number of medical men is consequently comparatively small; the ordinary members of the society publishing the volume before us amounts only to twenty-five. This society was founded in 1822, and we are happy to learn from the preface that it proceeds in its praiseworthy objects with undiminished vigour. The present collection contains twenty communications of some length, and forty-six miscellaneous notices of less pretensions. Many of these papers are very creditable to their authors, although several of them have become antiquated from the long period that has intervened between their presentation and publication. Others are of inferior value, and scarcely interesting to the public, although doubtless valuable at the time to the members of the society. We shall extract a few of the cases and shorter communications which appear to us the most inviting.

1. *Case of variola after vaccination, following scarlatina.* This case, related by Dr. Prevot, deserves to be recorded as an interesting anomaly, although we confess it might have been rendered more valuable had it described in detail the modifying influence of vaccination on the variolous disease.

The inhabitants of a farm-house, in a village five miles from Riga, were attacked with variola. The farmer, a young man twenty-two years of age, whose arm presented very distinct traces of vaccination, having every confidence in its prophylactic power, attended on the sick with the utmost assiduity for a period of fourteen days, till he was disabled by an attack of "scarlatina synochalis." The disease ran a favorable course. On the eighteenth day, the farmer being sufficiently recovered to leave his bed, put on a baize great coat which he had worn before his illness, and in which he had visited those in his house who had been attacked with variola. The next day he felt ill, and in the night grew feverish; the symptoms indicative of variolous infection then gradually appeared, and were succeeded on the fifth day by the eruption. The attendant fever was synochal. Recovery took place without any untoward occurrence.

The reflective reader of the preceding case will very naturally put the question, was the protecting influence of vaccination here destroyed by the occurrence of scarlatina, and the system thereby rendered susceptible of the variolous contagion?

2. *Diagnosis of phlebitis.* In a short essay on phlebitis, by Dr. Stracksen, three cases are recorded, which correspond in presenting the following symptoms, and in so prominent a manner as, in the opinion of the writer, to warrant their being considered diagnostic marks of the disease. We give them, since every contribution to the exact pathology of this fearful malady is deserving of notice, and may be useful as a guide in practice.

1. An indescribable restlessness and anxiety, seen in scarcely any other disease to the same extent.

2. Pulsation of the veins, and proportionately violent fever, with very frequent pulse, but softer than in arteritis.

3. Short and painful breathing, very different from that which attends pneumonia.

4. The fatal termination of the disease, often occurring on the fourth or sixth day, from complete disorganization, and consequent "paralysis" (loss of power) of the veins. (p. 174.)

3. *Remarkable case of abortion, by Dr. Bohlschwing.* A robust girl conceived in February, and in consequence menstruation ceased. In June she aborted. To her dismay, soon after, the symptoms of advanced pregnancy appeared, and in the beginning of November, five months after the abortion, she was delivered of a full-grown child, which doubtless was the result of the same impregnation, as the fœtus expelled at the fourth month. (p. 180.)

*Anomalous lactation, by Dr. Hartmann.* An infant whose mother was in good health, and had borne several children, exhibited a healthy appearance for the first five weeks after its birth. The alvine evacuations then became copious, fluid, and discoloured, and the child lost flesh and strength. After the usual remedies had been vainly administered for a fortnight, the mother remarked that the child did not take the right breast willingly, and so much did this unwillingness increase that at length the mere application of the nipple to the infant's lips occasioned loud crying. On examination it was found that the milk of the right breast had a distinctly *salt* taste, whereas the milk of the opposite breast was of the ordinary sweetness. No difference of consistence or colour was discoverable. From that time the child was only allowed to suck the left breast, and in a few days all diarrhœa and sickliness of appearance vanished. It is not a little singular that while the mammary secretion was thus unnatural, the health of the mother remained unimpaired. (p. 183.)

4. *Case of pain in the head cured by ol. terebinthinæ, by Dr. Mebes.* A single lady aged nineteen, previously healthy, was the subject of nervous fever. In the course of the disease, and particularly during recovery, there arose intense pain in the head, principally in the orbital and temporal regions, coming on suddenly, and as suddenly disappearing, and lasting ordinarily an hour, its longest duration being half a day. The pain subsequently ushered in each menstrual period. The lady married in her twentieth year, and shortly after became pregnant. The symptom which had so tormented her disappeared, but only to return as soon as the milk fever set in, when it continued with uninterrupted severity for several days. The child was now weaned. After leaving the lying-in room the patient suffered fresh and even longer attacks of pain, frequently of from eight to fourteen days' continuance. In eleven years she had borne five living children, and had miscarried twice; during *each pregnancy* the headach totally *ceased*, but no sooner was the lying-in chamber left than the pain returned, and was a constant attendant of menstruation. No relief was afforded by any plan of treatment, perhaps in consequence of the caution with respect to remedies supposed to be required by the frequent recurrence of pregnancy. Antarthritics, narcotics, antispasmodics, calomel to the extent of producing salivation, bark, and sea-bathing had no effect. At length *oil of turpentine* was tried in doses of ten drops, increased to half a teaspoonful every two hours, and continued for four months, and then gradually diminished. After pursuing this plan for some time, the patient entirely recovered. At first, the remedy was highly nauseating, but in large doses it produced an agreeable excitement. Its influence on the urinary secretion was very decided, but less so on the skin. (p. 185.)

5. *Case of consumption cured by ol. asphalti, by Dr. Mebes.* A woman presented the following group of symptoms: constant violent cough, with abundant purulent expectoration; night sweats, and frequent diarrhœa of a year's standing. She had several times expectorated blood, and had lost several grown-up children with hectic symptoms. The disease continued, the purulent expectoration increasing in spite of remedies, especially superacetate of lead. The oleum asphalti depuratum was then commenced, in three-drop doses three times a day, increased by a drop daily up to twenty drops. Manifest amendment took place from the fourth week; the cough and expectoration disappeared, and the strength returned. Goat's milk with Seltzer water completed the patient's recovery.

This case is incomplete, as the evidence from auscultation does not appear, and this is the more to be regretted, since cases of bronchitis simulating pulmonary phthisis often occur in practice, and it is in such instances that medicines of the bituminous class have often displayed their beneficial operation. (p. 186.)

6. *Remarkable formation of pediculi, by Dr. Von Wilpert.* A male infant, three months and a half old, was seized with symptoms of cerebral congestion with convulsions, on the subsidence of which an erratic form of roseola made its appearance. Scarcely had two days passed from the termination of the exanthema, when the child, who had recovered its good looks and tranquillity, became on a sudden extremely restless, throwing its head backwards, and as constantly moving its arms so as at first to favour the idea that the cerebral disturbance had recurred. On a closer survey, however, the irritation was found to arise from a quantity of small pediculi on the occiput, neck, and shoulders. This discovery was the source of much surprise, in consequence of the remarkable cleanliness of the family; besides, the infant's body had been regularly washed and bathed throughout the disorder, and not the smallest trace of vermin had ever been detected. A slight perspiration was observed on the head for the last day or two, which was followed in two days by this extraordinary parasitic development. The number of pediculi exceeded a hundred; in size, roundness, and whiteness, they resembled the pediculi that occasionally infest the human head as well as brutes. As soon as they were removed the child became quiet, and speedily recovered. (p. 200.)

7. *Case of ventral hernia, by Dr. Von Wilpert.* A woman, aged twenty-five, complained of severe abdominal pain four days after being delivered of her third child. The pain seemed *anterior to* rather than *in* the abdomen, and relief was sought from forcible pressure. A flaccid sacciform tumour was apparent below the umbilicus, and to this spot the pain was referred. Nothing but skin and peritoneum seemed to form the parietes of the tumour, which, the narrator was told, first showed itself after the second labour. The patient herself imagined that the extreme suffering which marked the end of this her third pregnancy, depended on the child being contained in the tumour, as to this point the general abdominal pain and tenderness were observed to converge. The treatment, though active, failed, and on the eighth day death closed the scene. A post-mortem examination disclosed the morbid appearances of peritonitis. The recti muscles were found separated in the linea alba, and pressed backwards so as to allow of a protrusion of peritoneum large enough to contain the gravid uterus and part of the intestines, which after delivery had evidently lodged in the hernial sac. A similar case of ventral hernia, whose contents were the gravid uterus, is recorded by Dr. Dahlhof in Rust's Magazine, Bd. 52. H. 1. (p. 201.)

